# Maternal exposures and the infant gut microbiome: a systematic review with meta-analysis

**DOI:** 10.1080/19490976.2021.1897210

**Published:** 2021-05-12

**Authors:** Allison Grech, Clare E Collins, Andrew Holmes, Ravin Lal, Kerith Duncanson, Rachael Taylor, Adrienne Gordon

**Affiliations:** a Central Clinical School, Faculty of Medicine and Health, University of Sydney, Camperdown, New South Wales (NSW), Australia; b Charles Perkins Centre, University of Sydney, Camperdown, NSW, Australia; c School of Health Sciences, Faculty of Health and Medicine, University of Newcastle, Callaghan, NSW, Australia; d Priority Research Centre for Physical Activity and Nutrition, University of Newcastle, Callaghan, NSW, Australia; e School of Life and Environmental Sciences, Faculty of Science, University of Sydney, Camperdown, NSW, Australia

**Keywords:** Pregnancy, infant gut microbiome, child health, women’s health, gut microbiome diversity, systematic review

## Abstract

Early life, including the establishment of the intestinal microbiome, represents a critical window of growth and development. Postnatal factors affecting the microbiome, including mode of delivery, feeding type, and antibiotic exposure have been widely investigated, but questions remain regarding the influence of exposures *in utero* on infant gut microbiome assembly. This systematic review aimed to synthesize evidence on exposures before birth, which affect the early intestinal microbiome. Five databases were searched in August 2019 for studies exploring pre-pregnancy or pregnancy ‘exposure’ data in relation to the infant microbiome. Of 1,441 publications identified, 76 were included. Factors reported influencing microbiome composition and diversity included maternal antibiotic and probiotic uses, dietary intake, pre-pregnancy body mass index (BMI), gestational weight gain (GWG), diabetes, mood, and others. Eleven studies contributed to three meta-analyses quantifying associations between maternal intrapartum antibiotic exposure (IAP), BMI and GWG, and infant microbiome alpha diversity (Shannon Index). IAP, maternal overweight/obesity and excessive GWG were all associated with reduced diversity. Most studies were observational, few included early recruitment or longitudinal follow-up, and the timing, frequency, and methodologies related to stool sampling and analysis were variable. Standardization and collaboration are imperative to enhance understanding in this complex and rapidly evolving area.

## INTRODUCTION

### The developmental origins of health and disease and the microbiome

The Developmental Origins of Health and Disease (DOHaD) hypothesis proposes that the period from conception to age 2 years – the ‘First 1,000 Days’ of life – is a critical window of growth and development.^1^ This period is characterized by rapid maturation of the metabolic, endocrine, neural, and immune signal/response pathways that underpin regulatory capabilities – all of which are highly influenced by both the maternal milieu and exposures in the perinatal environment. Collectively, these can ‘program’ offspring development in ways that affect future disease risk.^2,3^ During these first thousand days, fetal exposure to microbial metabolites occurs and ultimately microbial colonization of the neonatal gastrointestinal tract also takes place as an integral part of postnatal maturation.^4,5^ The outcome is a complex adaptive system (human ‘holobiont’) with an interconnected network between microbiome, immune function, and metabolic regulation, affecting an individual’s nutrient metabolism, development, and long-term health outcomes.^2,3,6,7^ The DOHaD framework may therefore be extended to incorporate the microbiome (a dynamic community with the property of self-similarity over time), in recognition of the influence of perinatal factors on microbiome assembly in early life, and their possible long-term effects.^7^

The presence and activity of a “symbiotic” intestinal infant microbiome is known to affect processes of nutrient uptake, maintenance of gut barrier function, and immune response ^8^ as well as promoting optimal growth, ^2,3,9^ immune system maturation, ^10,11^ and neurodevelopment.^12,13^ Such wide-ranging influence means that maladaptive interactions between the nascent postnatal microbial community, host, and their environment are also possible and a spectrum of adverse outcomes, including obesity, inflammatory bowel disease, allergies, asthma, cardiovascular disease, and neurodevelopmental disorders have been associated with microbiome differences.^14,15^ Whilst only part of the complex interplay between a host and their genetics and environment (much of which is genetically constrained), it follows that any factor that influences the composition, stability, or activity of the microbial community (or affects the host capability to perceive and respond) in early life may be considered a risk factor for predisposition toward disease. The concept of “dysbiosis” has been proposed to distinguish those diseases with complex etiology that cannot be adequately explained by genetic factors or a single pathogenic agent. Observational studies suggest that gestational age, ^16–18^ delivery mode ^16,19–24^ and early postnatal factors, including infant feeding, ^21,25,26^ antibiotic treatment, ^27,28^ and household exposures, ^29–32^ affect the composition and/or diversity of the infant intestinal microbiome. However, differences in sample size and methodologies concerning how and when the microbiome is characterized, and indeed the application of the very concepts of microbiome and dysbiosis, have limited comparison of findings across studies.

### ‘Normal’ development of the human intestinal microbiome

In order to measure the effect of exposures on shaping the infant microbiome, the determination of a ‘normal’ infant microbiome state is a crucial, yet challenging task. There has been significant variation in how the characterization of the microbiome has been performed, ^33,34^ alongside a growing appreciation for potentially vast differences between the microbiomes of apparently healthy people that underpin unique and complex interactions with their environment, genetics, and lifestyle.^35,36^ Likewise, understanding the expected development and maturation of an individual’s gut microbiome from birth is ongoing. Whilst controversy remains regarding antenatal exposures such as *in utero* gut colonization ^37–39^ and the long-term influence of perinatal exposures including delivery mode, ^40,41^ it is understood that children under 36 months of age have dynamic and highly individual microbial profiles characterized by a lower diversity index (fewer bacterial species) compared to older children and adults.^42^ Postnatal development of an individual’s microbiome appears to occur in two colonization ‘phases’, separated by the introduction of solid food around 6 months, and is further influenced by illness, antibiotic exposure, and other environmental factors (Table 2). The interplay between these exposures informs the establishment of a comparatively stable, “adult-like” composition by early childhood, ^43,51,52,58,59^ including those born prematurely.^60^ Preterm neonates have a less species-diverse microbiome, often with an abundance distribution characterized by higher levels of facultatively anaerobic pathogens such as *Enterobacter, Enterococcus, Klebsiella*, and *Staphylococcus* compared to term neonates.^61,62^ These differences persist until children are at least 4 years of age.^62^

**Table 1.** Summary of included publications according to major characteristics

**Figure:** 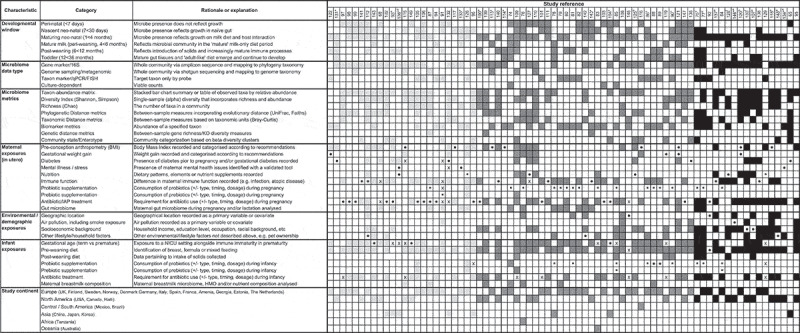

**Table 2.** Summary of typical development of the intestinal microbiome from birth to early childhood

Stage of development	General microbiome characteristics (reference/s)
Neonate (0–4 weeks)	Rapid colonization by anaerobic bacteria.^43,44^ Meconium is likely to be more reflective of maternal microbiome composition than in response to the delivery mode or environmental conditions.^43–45^ Altered pattern of colonization associated with prematurity; preterm infants have higher counts of *Enterobacteriaceae* and lower counts of *Bacteroidaceae* and Bifidobacteria compared to those born at term.^46,47^ Lower species diversity was also observed in preterm compared to term neonates.^48^ Differences in microbiota composition due to birth mode are present.^49^ Infants born vaginally are characterized by a higher relative abundance of Bacteroides whereas those born via cesarean section delivery have a lower relative abundance of *Bifidobacterium* and higher levels of *Klebsiella, Haemophilus, and Veillonella*.^50^
Milk-fed infant (0–6 months)	Overall low species diversity continues.^21^ Differences in individual species abundance differ by feeding type (breastmilk or formula).^49^ Highly individual composition (i.e., high beta diversity).^49,51^ *Bifidobacterium* is the dominant bacterial genus, particularly in breastfed infants.^52–54^
Mixed-fed infant (6–12 months)	Introduction of solid food spearheads change in composition, including an increase in the relative abundance of *Bacteroides* and newly dominant genera such as *Ruminococcus and Akkermansia*.^43–45^ Cessation of breastfeeding may or may not occur, has a major impact on composition independent of the introduction of solid foods.^49,55^ Increasing alpha diversity (Shannon Index) driven largely by increasing species richness.^49,50,53^ Highly individual composition (beta diversity) persists.^49,51^
Toddler (12–36 months)	Increasing phylogenetic diversity and reducing beta diversity.^51^ Cessation of breastfeeding has a major impact on microbiota composition and alpha diversity alongside increasing dietary diversity.^49^ Approaching microbiome ‘stability’ with ongoing adaptation to the environment.^42,51,53^
‘Adult-like’ microbiota (>36 months)	Microbiome is more resilient to environmental challenges.^56^ Stable microbiota ‘signature’ is established.^42,51,53^ Alpha diversity (Shannon Index) in early childhood continues to be lower than that of adults.^56,57^

Shortly after birth, the neonatal gut is rapidly colonized by facultatively anaerobic bacteria typically including strains of *Enterobacter, Enterococcus, Staphylococcus*, and *Streptococcus* genera.^4,5^ Colonization continues with the commencement of milk feeding, and the development of a simple community of obligate anaerobes, though breastfed infants have distinct microbial colonization patterns compared to their formula-fed counterparts, ^4,26,63,64^ further modified by breastfeeding exclusivity and duration.^26^ Results from the Canadian Healthy Infant Longitudinal Development (CHILD) study showed that the richness and diversity of infant gut microbiota at 4 months of age are lowest in exclusively breastfed infants, higher in partially breastfed infants, and highest in infants receiving formula.^64^ Low gut bacterial diversity in breastfeeding infants is thought to be a result of breastmilk oligosaccharides which serve as substrates for a limited number of gut microbes.^65^ Meanwhile, infants who are predominately formula-fed are associated with lower levels of *Bifidobacteria* and *Lactobacilli* alongside higher diversity, dominated by Bacteroides, *Staphylococci, Enterococci*, and *Clostridia*.^5,21^ At 12 months, there remain distinct characteristics in microbiome composition in infants still receiving breastmilk, characterized by enrichment of *Bifidobacteriaceae, Veillonellaceae* and Proteobacteria.^64^ Whether early differences in microbiome composition owing to feeding type persist across early childhood is less clear. A recent follow-up study found no significant differences in microbiome composition at 1, 2, or 4 years of age according to breastfeeding in infancy.^62^

With the introduction of solid foods around 6 months of age, the infant microbiome evolves from a simple, *Bifidobacteria*-rich community to a more diverse community structure alongside increased dietary diversity.^4,5^ Although the introduction of solid food spearheads these changes, at 12 months, breastfed infants have a greater relative abundance of *Bifidobacterium* and *Lactobacillus* species compared to that fed formula.^21^ Cessation of breastfeeding, rather than exposure to solid food, appears to be associated with maturation toward an “adult-like” microbiota.^4,21,42^ Crudely dominated by Bacteroidetes and Firmicutes and with increased alpha diversity, ^4,5^ this mature microbial profile is more resilient to environmental challenges ^66–68^ but remains capable of adaptive shifts in community structure well into childhood.^53,56,57,62^

***Links between prenatal maternal exposures, postnatal factors and infant gut microbiome assembly: what do we know and where are the knowledge gaps?***

Pregnancy is accompanied by changes associated with metabolic dysfunction, including insulin resistance, dyslipidemia and hypertension, though whether these physiological changes are driven by intestinal microbiome shifts in the mother, and the consequences of these shifts on the developing fetus and their subsequent microbiome, are less clear.^69,70^ Maternal nutrition has been shown to play a key role in developmental programming and modification of non-communicable disease risk in offspring via epigenetic changes.^71^ Other maternal factors, including antibiotic use, diet, obesity, and diabetes have been associated with altered development of their children’s intestinal microbiome.^72–75^ Further, although a metabolically active microbial community is not present in the gut until after birth, some microbe exposure may occur *in utero*, including microbe-derived molecules produced by the mother’s own microbiome, which may have an early predisposing effect signaling pathways in her child.^2^ Postnatally, questions remain regarding normal or ‘optimal’ intestinal microbiome assembly and consequences of disturbances to this sequence, ^37–39^ and it remains challenging to isolate the impact of advancing age, environmental exposures, and concomitant gut maturation from the direct effects of diet and other factors.^76^ Previous narrative reviews have outlined perinatal influences on infant microbiome development, ^10,63,77,78^ though the strength and nature of associations have not been systematically evaluated and many underlying mechanisms remain unclear.

The aim of this work was to systematically review, and where possible to conduct a meta-analysis on the evidence for relationships between maternal exposures during pregnancy and measures of the infant intestinal microbiome. Secondary aims were to identify knowledge gaps, highlight the diversity of methods used in the field, and provide recommendations for future research to better understand mechanistic links between exposures, alterations in microbiome composition, diversity and/or function, and later-life disease risk.

## METHODS

This systematic review protocol was registered with PROSPERO Online ^79^ [CRD42020150602] and was conducted according to the Preferred Reporting Items for Systematic Reviews and Meta-Analysis (PRISMA) statement.

### Study identification

The literature search was conducted by AMG. Studies published in English from five relevant databases (MEDLINE, EMBASE, Scopus, CINAHL, Cochrane/CENTRAL) were collected in August 2019, using identified keywords and index terms. The search terms were divided into three groups, combined with the Boolean phrases “AND” between groups and “OR” within groups: ^1^ Pregnancy/or pregnan*.mp., or Prenatal.mp., or Perinatal.mp., or Maternal.mp., or Mother*.mp. and ^2^ Infant.mp. or Infant/or Infant health.mp. or Infant Health/or birth outcome.mp. and ^3^ Microbiome.mp. or Microbiota/, or gut microbiome.mp. or Gastrointestinal Microbiome/. There were no limits applied regarding the year of publication.

### Inclusion and exclusion criteria

Articles were included if they: ^1^ were intervention or observational studies (randomized controlled trials, case-control, cross-sectional or longitudinal studies) conducted in humans only, ^2^ collected pre-pregnancy or pregnancy ‘exposure’ data, independent of the maternal intestinal microbiome (any kind of exposure permitted), from which an impact on infant outcomes was assessed, and ^3^ collected infant outcome/s, including at least one measure of the infant intestinal microbiome at less than or equal to 12 months of age.

Articles were excluded if they: ^1^ were of an inappropriate study type (animal studies, in vitro studies, conference abstracts, and proceedings published more than 2 years ago, or which have subsequently resulted in a published paper [in which case, the paper was included, if appropriate], case series, case reports, book chapters, guidelines, commentaries, editorials, letters to the editor, reviews and meta-analyses), ^2^ did not collect pre-pregnancy or pregnancy “exposure” data, and/or ^3^ did not measure infant intestinal microbiome composition or diversity beyond identifying the abundance of a specific species only.

### Study selection

All studies identified were retrieved from online databases and exported to the reference management system, EndNote (Version X8, Thomson Reuters, New York, NY, USA). Studies were screened by AMG, based on titles and abstracts. Full texts were retrieved following screening and subsequently reviewed by AMG and three other authors (RL, RT, and KD). Disagreements were resolved by discussion with a third investigator (AG).

### Study quality

AMG evaluated the methodological quality of included studies using the Scottish Intercollegiate Guidelines Network (SIGN) checklists ^80^ for cohort and case–control studies and controlled trials. Studies were assessed on their internal validity with respect to subject selection, outcome assessment, confounding, and statistical analysis. Overall quality was coded as high, acceptable, or low/unacceptable based on responses to questions in the SIGN tool (see Supplementary Table 1 for assessments for each included study).

### Data extraction and synthesis

Data relating to study design, sample size, participant demographics, pregnancy “exposure/s”, infant outcomes, measurement of the infant microbiome, and recruitment and follow-up periods were extracted using a pre-determined template on Microsoft Excel. This information was used to construct Table 1 and Supplementary Table 1, according to the major study characteristics identified that related to the microbiome.

For Table 1, articles were categorized by study type (intervention or observational, denoted with a square or circle symbol, respectively) and this symbol was used to identify the primary “exposure” of interest in that publication. Following this, sample size (*n*) was categorized based on the interquartile range of *n* for included studies rounded to the nearest 50, generating the following three categories: 0–50 (“small study”, light gray), 50–300 (“medium study”, dark gray), and 300 or more (“large study”, black). These shades were then applied to each article characteristic, including study features (study continent and microbiome testing timing, methodology, and metrics), and both maternal and infant features. Secondary “exposure” outcomes (if measured) were also shaded according to study size. Finally, articles were stratified according to sample size (small to large, from left to right).

For each included publication, data pertaining to the composition and/or diversity of the infant intestinal microbiome were collected, according to the metrics used by authors. In studies investigating the impact of maternal probiotic supplementation, the presence or absence of the species provided by the probiotic supplement was also compiled (Table 3).

**Table 3.** Summary of included articles investigating the impact of probiotic supplementation during pregnancy on measures of the intestinal microbiome composition and diversity in their infants (n = 17). Where multiple articles have been published within the same study cohort, these have been grouped together

Author, year	Probiotic	Administration and timing	Infant age	Impact of probiotic supplement on infant microbiome composition and/or diversity
Abrahamsson 2009 ^81^	*Lactobacillus reuteri*	Mothers: daily from 36 weeks’ gestation until delivery. Infants: daily from 1–3 days until 12 months of age.	5 days 1 month 3 months 6 months 12 months	*Lactobacillus reuteri* prevalence was significantly higher in the probiotic group compared to placebo at 5 days of age detected in 82% vs 20% (*P* < .001). After this, the prevalence of *Lactobacillus reuteri* declined despite continuous supplementation in infants and a high compliance rate.
Avershina 2016,^82^ Dotterud 2015 ^83^	*Lactobacillus rhamnosus* GG, *Bifidobacterium animalis subsp. lactis* Bb-12, and *Lactobacillus acidophilus* La-5	Mothers: daily from 36 weeks’ gestation until 3 months postpartum.	10 days 3 months 12 months	*Lactobacillus rhamnosus GG* colonized infants in the probiotic group with greater relative abundance at 10 days (*P* < .005) and 3 months (*P* < .005) of age, compared to controls. This difference was not maintained at 12 months (*P* = .783) or 2 years (*P* = .511). No statistically significant differences were found between the probiotic and placebo groups in the a- or b-diversity of the total microbiota in infant stool samples at 3 months or 2 years of age.
Bisanz 2015 ^84^	*Lactobacillus rhamnosus* GR-1	Mothers: daily from 12–24 weeks’ gestation until 1 month postpartum.	3 days 1 week 1 month	Infants aged 10–25 days whose mothers received probiotics had a 3-fold increase in relative abundance of *Bifidobacterium* and a 16.8-fold decrease in *Enterobacteriaceae* (*P < *.05), compared to controls.
Enomoto 2014 ^85^	*Bifidobacterium longum* BB536 and *Bifidobacterium breve* M-16 V	Mothers: daily from 36 weeks’ gestation until delivery. Infants: daily from 1 week until 6 months of age.	4 months 10 months	At 4 months of age, the relative abundance of Bacteroidetes was significantly higher in the microbiota of infants in the probiotic group compared to the control group (*P* = .025). No difference was measured in the stool samples obtained at 10 months of age (*P* = .770).
Grönlund 2007,^86^ Grönlund 2011 ^87^	*Lactobacillus rhamnosus* with *Bifidobacterium longum* (or) *Lactobacillus paracasei* with *Bifidobacterium longum*	Mothers: daily from 15 weeks’ gestation until 6 months postpartum or cessation of exclusive breastfeeding.	1 month 6 months	Correlations between mothers’ and infants’ fecal Bifidobacterial counts determined by quantitative real-time PCR were not affected by probiotics during supplementation at 1 month (*P* = .11 for Bifdobacterium, *P* = .40 for *B. longum*) but were significant at 6 months, after probiotics were ceased (*P* = .043 for Bifidobacterium, *P* = .023 for *B. longum*). Bifidobacterial diversity indexes were not significantly different between infants in probiotic or control groups (P not provided).
Grześkowiak 2012 ^88^	*Lactobacillus rhamnosus* with *B. longum strain BL999* (or) *Lactobacillus paracasei* with *B. longum strain BL999*	Mothers: daily from 2 months before delivery until 2 months postpartum.	6 months	At genus level, Bifidobacterium counts were significantly different among the study groups (*P* = .017), lowest in the *Lactobacillus rhamnosus* group compared to placebo. The relative abundance of Bifidobacterium was not significantly different between groups (*P* = .642). In infants whose mothers were supplemented with *Lactobacillus rhamnosus* compared to placebo, there was a significant difference in the relative abundance of the *Lactobacillus-Enterococcus* group (*P* = .003). No other statistically significant differences were found between probiotic and placebo groups regarding the relative abundance of major bacterial groups studied (*Prevotella, Clostridium histolyticum* and *Akkermansia muciniphila*).
Gueimonde 2006 ^89^	*Lactobacillus rhamnosus* GG	Mothers: 2–4 weeks before delivery until 3 weeks postpartum. Frequency of delivery not further specified.	5 days 3 weeks	At 5 days of age, infants whose mothers received *L. rhamnosus GG* were significantly more likely to be colonized with *B. breve* (*P* = .044) compared to the placebo group. This effect did not persist until 3 weeks (*P* = .069) and there were no other significant differences between groups for other Bifidobacterium species tested. *L. rhamnosus GG* supplementation in mothers did not significantly increase gut bifidobacterial diversity in infants at 3 weeks (*P* = .134).
Ismail 2012 ^90^	***Lactobacillus rhamnosus* GG**	**Mothers: daily from 36 weeks’ gestation until delivery.**	**7 days**	**Prenatal supplementation with *Lactobacillus rhamnosus* GG had no significant effect on fecal microbial diversity in 7 day old infants, assessed using terminal restriction fragment length polymorphism using restriction enzymes *Sau*96I and *Alu*I (*Alu*I 14.4 vs. 15.5, *P* = .17, 95% CI −0.4, 2.5; *Sau*96I 17.3 vs. 15.8, *P* = .15, 95% CI −3.5, 0.5).**
Korpela 2018 ^91^	*Bifidobacterium breve, Propionibacterium freundenreichii* subsp. *shermanii* JS, *Lactobacillus rhamnosus* and *Lactobacillus rhamnosus* GG. Infants 1.supplemented with the same probiotic and prebiotic galactooligosaccharides	Mothers: daily from 35 weeks’ gestation until delivery. Infants: daily from birth until 6 months of age.	3 months	The probiotic supplement had a strong overall impact on the microbiota composition, but this depended on the infant’s diet, and the specific effect of maternal probiotic supplementation was not possible to measure. In breastfed infants, those supplemented with probiotics had a greater relative abundance of Lactobacilli (100%) and Bifidobacteria (29%) compared to controls (*P* < .0001). Other taxa were reduced in abundance in the probiotic group: Clostridia by 66% (*P* < .0001) and Gammaproteobacteria by 58% (*P* < .0001). In formula-fed infants, the total abundance of Bifidobacteria was slightly but significantly decreased in those supplemented with probiotics (by 7%, *P* < .0001). Several Firmicutes and Proteobacteria taxa were also increased in the formula-fed supplemented group compared to the formula-fed control group: Anaerostipes by fourfold (*P* = .05), Veillonella by sevenfold (*P* < .0001) and Klebsiella by sixfold (*P* = .05).
Kukkonen 2007 ^92^	*Lactobacillus rhamnosus* GG, *L rhamnosus, Bifidobacterium breve* and *Propionibacterium freudenreichii* ssp. *Shermanii* JS Infants supplemented with the same probiotic and prebiotic galactooligosaccharides	Mothers: twice daily from 36–38 weeks’ gestation until delivery. Infants: once daily from birth until 6 months of age.	Meconium 3 months 6 months	At 3 and 6 months, the probiotic group was significantly more frequently colonized with Lactobacilli and Propionibacterium (*P* < .001). Fecal counts of total Bifidobacteria (*P* = .001) and Lactobacilli (*P* < .01) were significantly higher at 6 months. At 2 years, no differences were observed between study groups in fecal bacterial colonization.
Niers 2009,^93^ Rutten 2015 ^94^	*Bifidobacterium bifidum, Bifidobacterium lactis* and *Lactococcus lactis*	Mothers: daily from ~32 weeks’ gestation until delivery. Infants: daily from birth until 12 months of age.	1 week 2 weeks 1 month 3 months 12 months	At time of supplementation, probiotic species had a higher abundance and prevalence in the probiotic group, but this difference was not maintained after supplementation was stopped. Bifidobacteria were significantly higher in the probiotic group at 1 month of age (*P* = .003) and *Lactococcus lactis* was significantly higher at 2 weeks (*P* = .001) and 1 month of age (*P* = .03). *Lactococcus lactis* was absent in the placebo group during the intervention period and was significantly higher in abundance at two years (*P* = .01).
Parnarnen 2018 ^95^	*Lactobacillus rhamnosus* LPR and *Bifidobacterium longum* (or) *Lactobacillus paracasei* and *Bifidobacterium longum*)	Mothers: daily from 2 months before and 2 months after delivery.	1 month 6 months	No significant changes were observed in the abundance of antibiotic resistance genes in the infants between probiotic and placebo groups.
Rinne 2005,^96^ Rinne 2006 ^97^	*Lactobacillus rhamnosus* GG	Mothers: daily from 36 weeks’ gestation until delivery. ± Infants: daily from birth until 6 months of age.	3 months 6 months 12 months	Total bacterial counts in fecal samples showed a decreasing trend from 3 to 12 months of age (*P* < .0001) which were not different between probiotic and placebo groups (*P* = .70). At 6 months, there were less Clostridia in feces in the placebo compared with the probiotic group (*P* = .026). No other statistically significant differences were found between placebo and probiotic groups at 6 months in Bifidobacterium (*P* = .145), Bacteroides (*P* = .882), Lactobacillus/Enterococcus (*P* = .817) and total bacterial counts (*P* = .125).

### Statistical analysis with meta-analysis

It was not possible to perform meta-analyses for all exposure groups, owing to diverse interventions and/or outcomes measured with respect to the infant gut microbiome. Associations were described as significant if *P* < .05. For exposures where meta-analysis was possible, data were pooled according to pregnancy exposure using a random-effects model in Review Manager Software, version 5.4 (RevMan, 2020). The Shannon Index, a measure of within-sample (alpha) diversity, based on the sum of the proportion of each species relative to the total number of species in the community, ^98^ was used as the primary outcome on which study outcomes were compared in meta-analyses. Unless otherwise stated, alpha diversity was reported using the Shannon Index, calculated on taxon abundances after classification to species rank. Authors were contacted if data were not available in the text; otherwise, data were calculated from included figures. Where data were not available, the publication was not included in the meta-analysis. Means and standard deviation (SD) were used to calculate the mean difference (MD) in alpha diversity between groups, according to pregnancy exposure. Heterogeneity was assessed in each meta-analysis using Chi^2^, degrees of freedom (df), and I^2^ statistics. Using the I^2^, heterogeneity was regarded as moderate to substantial if 30–75% and considerable if greater than 75%, based on Cochrane recommendations.^99^

## RESULTS

### Study selection

**Figure 1.**
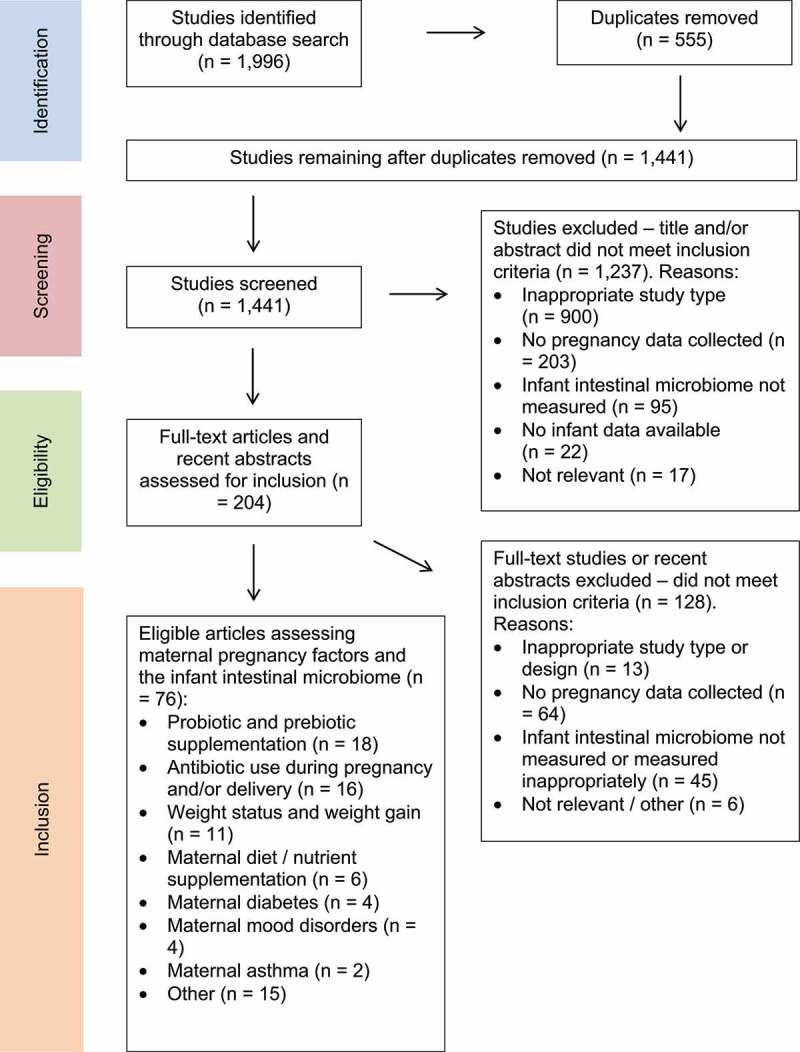
Preferred Reporting Items for Systematic Reviews and Meta-analysis (PRISMA) flow diagram of the study selection process for the current review on maternal exposures during pregnancy and associations with the infant intestinal microbiome

Study selection is summarized in Figure 1. The initial database search identified 1,441 unique publications after the removal of duplicates. Further 1,237 publications were excluded after assessing titles and abstracts based on study type, missing data, and/or irrelevance. Full texts of 204 articles were retrieved. At this stage, articles were excluded if they did not measure a specific pregnancy “exposure” compared to an infant outcome (n = 64) or if the measurement of the infant intestinal microbiome was limited to one species only and/or a valid methodology was not used (n = 45). Others were excluded based on inappropriate study type (n = 3) or other reasons (n = 6). A total of 76 publications were therefore included in the review.

### Study characteristics and methodologies

The characteristics of included studies are summarized in Table 1. Detailed information regarding each included publication is outlined in Supplementary File Table 1. Studies were published from 2004 to 2019, with the majority (72%) from the past 5 years (2015–2019). Most studies were observational (74%, n = 56). The remaining 20 articles (26%) were intervention studies, 12 of which were randomized controlled trials. The settings of included studies were Europe (n = 39), North America (n = 26), Asia (n = 8), Africa (n = 1), South America (n = 1), and Oceania (n = 1).

The total number of mother-infant dyads included in the review was 17,509, with a median sample size of 84 (range: 6–1,681). Some studies purposefully recruited women with a history of allergic or other diseases (21%, n = 16). The majority of studies (71%, n = 54) recruited women of reproductive age via antenatal clinics, typically in their second trimester or later. Few (3%, n = 2) used advertising campaigns, or recruited via schools and shops (n = 1), and others recruited infants at or shortly after birth and retrospectively gathered maternal data (18%, n = 14). Five studies (7%) did not report a clear recruitment strategy. Body Mass Index (BMI) was the only exposure for which pre-pregnancy data were available in this review; all others related to factors during pregnancy and/or delivery.

### Study methodologies – stool microbiome collection and assessment

The majority of studies (76%, n = 58) used 16S rRNA gene sequencing as their primary methodology for the assessment of the infant microbiome, with 12 studies (16%) using more than one method (see Table 1 for further detail). Polymerase chain reaction (PCR) (16%, n = 12), fluorescence in situ hybridization (FISH) (11%, n = 8), and culture-based methods (4%, n = 3) were also used to quantify specific target populations. A small number of recent studies (9%, n = 7) also used metagenomic sequencing to further characterize the infant microbiome. Since all methods ultimately relied on a common rRNA phylogeny-based classification scheme, species composition and abundance distributions across studies were considered broadly comparable. Direct comparison of taxon abundances across different methods was not possible and readers are referred to Table 1 and Supplementary Table 1 to identify such cases.

The frequency and timing of stool sample collection and analysis also varied (Table 1). The mean number of stool samples analyzed per study was two; whilst five studies analyzed more than five samples, 93% sampled between one and five times across infancy. Almost half of the included studies sampled at least once within the first week of life (46%, n = 35), though 83% (n = 63) of studies sampled at least once between 1 and 4 months. Fewer studies sampled between 4 and 6 months (30%, n = 23) or beyond 6 months (16%, n = 12).

### Results by exposure variable

#### 3.4.1 Probiotic or prebiotic supplementation during pregnancy

Seventeen intervention studies ^81–97^ investigated the relationship between prenatal maternal probiotic supplementation and the infant intestinal microbiome (Table 3), with a variety of other outcomes studied (e.g., allergy development). Probiotics including one ^81,84,89,90,96,97^ or more ^82,85,83,86–88,91–95^ *Lactobacillus* and/or *Bifidobacterium* species were administered in the form of capsules/powder, ^85–95^ milk/yogurt ^82–84^ or oil droplets ^81^ to women in their second ^84,86,87^ or third trimester.^81–83,85,88–93,95–97^ Four studies did not describe randomization procedures with regard to probiotic intervention, ^85–87,89^ whilst all others were randomized controlled trials (Supplementary Table 1). Thirteen articles focused on allergy development in high-risk infants, recruiting pregnant women with a personal or family history of atopic disease, ^81,86–97^ and three publications investigated whether probiotic intervention during pregnancy and infancy affected the risk of allergy development.^85,92,93^ Here we focus on evidence of whether probiotic administration induced a measurable change in the infant microbiome.

There was limited evidence that maternal probiotic supplementation during pregnancy led to ongoing colonization by the probiotic strain within the infant gut microbiome or that it impacted overall infant microbiome diversity. As shown in Table 3, eight studies ^81,83–85,89,91,92,94^ found that at the time of probiotic intervention and shortly after birth, the species given as a supplement to the mother was detected at a significantly higher abundance in infants whose mothers received the supplement compared to controls. However, six studies ^82,85,86,93,97,94^ showed no significant differences between groups with respect to the relative abundance of supplemented species or microbiome diversity once the probiotic intervention was ceased. A further six studies ^81,85,91–93,97^ extended probiotic supplementation to include infants. Three publications ^87,92,93^ reported that probiotic supplementation to both mothers and infants significantly reduced the risk of eczema development (*P* = .007 at 10 months and *P* = .033 at 18 months; *P* = .035 at 24 months; and *P* = .035 at 3 months, respectively). However, the impact of *maternal* probiotic use on infant microbiota or allergy risk specifically could not be isolated with this study design.

Notably, 13 articles involved administration of preparations that included *Lactobacillus rhamnosus* strains, either as a single species probiotic ^84,89,90,96,97^ or in combination with a *Bifidobacterium* species, ^82,83,86–88,91,92,95^ to mothers and/or infants (Table 3). In one study, *Lactobacillus rhamnosus* GG given to mothers colonized infants in the probiotic group with greater relative abundance at 10 days (*P* < .005) and 3 months (*P* < .005) of age, compared to controls, though this difference was not maintained at 12 months (*P* = .783).^83^ Another study reported significantly higher total Bifidobacterial counts in supplemented compared to control groups at 6 months (*P* < .001); though both mothers and infants were supplemented.^92^ Otherwise, limited statistically significant differences were found between probiotic and placebo groups in terms of the relative abundance of Bifidobacterial strains or Bifidobacterial diversity between 5 days and 6 months of age.^86–89,96,97^ Overall diversity at 1 week of age and measures of alpha and beta diversity at 3 months of age were also not significantly associated with *Lactobacillus rhamnosus* supplementation in pregnancy.^83,90^

One study provided prebiotic supplementation (galactooligosaccharides and long-chain fructooligosaccharides) to pregnant women ^100^ and two studies provided prebiotics alongside probiotics to infants.^91,92^ The former study by Shadid and colleagues found that whilst the percentage of *Bifidobacteria* was significantly higher in mothers in the prebiotic group compared to the placebo group (*P* = .026), this was not transferred to neonates.^100^ *Bifidobacteria* and *Lactobacilli* diversity and similarity indexes, and the percentage of neonates who were positive for specific *Bifidobacterium* and *Lactobacillus* species, did not differ between prebiotic-supplemented and placebo groups across the first 6 months of life.^100^

Overall, results showed no persistent effect of maternal probiotic or prebiotic supplementation on the infant gut microbiome following cessation of treatment.

#### 3.4.2 Antibiotic use during pregnancy and delivery

**Figure 2.**
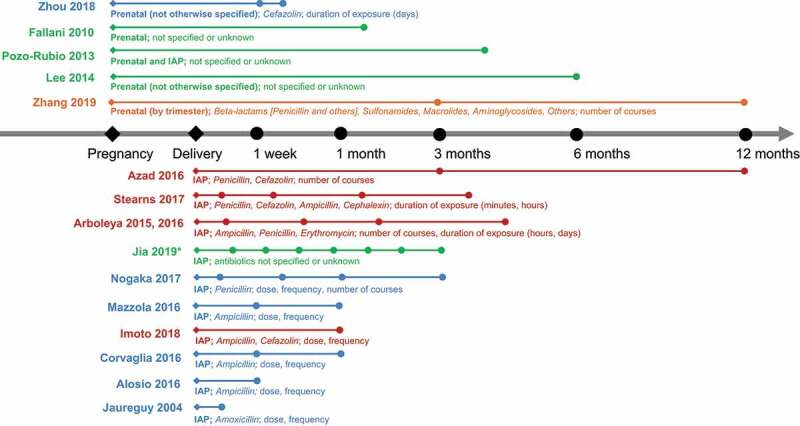
Timeline of stool sample collection across included articles investigating the role of prenatal antibiotic exposure or intrapartum antibiotic prophylaxis (IAP) on infant microbiome composition and/or diversity. Abstracts are denoted with an asterisk (*). Filled diamonds (◆) represent the time at which maternal antibiotic exposure occurred. Filled circles (∙) represent approximate times at which infant samples were collected (timeline not to scale). Articles investigating the use of a single antibiotic are denoted in blue, multiple antibiotics in red, and antibiotic categories in orange. Articles for which the type of antibiotic used was not specified are indicated in green. Type of antibiotic and measures of dose, frequency or duration of use are indicated for each study, where possible

Sixteen articles primarily explored the effect of oral antibiotic use in pregnancy ^101–105^ or intravenous antibiotic exposure during delivery (intrapartum antibiotic prophylaxis, IAP) ^48,106–115^ on infants’ intestinal microbiome composition and/or diversity (Figure 2). IAP was administered for maternal Group-B *Streptococcus* (GBS) positivity, ^106,108–111,113–115^ preterm premature rupture of the membranes (PPROM), ^108,110^ and/or as routine practice in caesarean-section deliveries.^108,110^ Five studies investigated antibiotics alongside other exposures.^116–120^

All included studies found some significant differences between the gut microbiome composition of infants whose mothers were exposed to antibiotics prenatally ^101−105^ or as IAP, ^48,106–115^ compared to unexposed infants (controls). In eight studies, a significantly reduced relative abundance of Actinobacteria, ^106,107,114^ specifically the *Bifidobacterium* genus, ^105,106,109,110,109,113,109,115^ was observed in infants whose mothers were treated with antibiotics prenatally or during delivery, compared to controls. In three studies, this difference did not persist beyond 30 days of age, by which time the *Bifidobacterial* population appeared to have recovered.^109,113,114^ The relative abundance of *Bacteroidetes* ^101,106^ was also significantly less in infants up to six weeks of age whose mothers were treated with antibiotics compared to controls. Meanwhile, eight publications reported an increased relative abundance of *Firmicutes* ^105,108,112–115^ and *Proteobacteria* ^106–108,113–115^ in infants following maternal antibiotic exposure. In contrast, another study found the relative abundance of *Firmicutes* was significantly higher in premature antibiotic-unexposed infants compared to those exposed to IAP (P < 0.01).^107^

Delivery mode (vaginal versus caesarean-section) modified the effect of antibiotic exposure in seven studies, ^48,104,105,101,104,105,108,104,105,110,104,105,115^ four of which found significant differences in infant microbiome composition between those exposed to maternal antibiotics and controls, independent of a “caesarean-section effect”.^104,108,110,115^ Azad and colleagues ^108^ found that the duration of differences between groups owing to delivery mode diverged between elective and emergency caesarean-section deliveries at 12 months; whilst infants exposed to IAP born via emergency caesarean-section were associated with a lower relative abundance of Bacteroidetes (P < 0.001) and a higher relative abundance of Firmicutes (P < 0.001) and Proteobacteria (P < 0.05) compared to vaginally-delivered infants without IAP exposure, no persistent microbiota differences were found among infants exposed to IAP who were born via vaginal or elective caesarean-section delivery.

Whilst information regarding the timing, duration, and frequency of antibiotic exposure was variably collected in included studies (Figure 2), the specific impact of timing and duration of antibiotic exposure was further explored in two studies.^104,115^ After adjusting for confounders, one study found that compared to unexposed controls, infants whose mothers used antibiotics in their second trimester of pregnancy had significantly different relative abundances of 13 and 17 bacterial amplicon sequence variants (ASVs) at three and 12 months of age, respectively.^104^ Stearns and colleagues ^115^ found that a longer duration of IAP had a greater negative effect on Bifidobacterium populations in 3-month-old infants, where every hour of maternal IAP was associated with a 7% decrease in the relative abundance of *Bifidobacteria*.

Differences in the gut microbiome diversity of infants exposed to prenatal antibiotics or IAP compared to unexposed infants were investigated in eight studies.^102,105,106,105,108,105,110,105,113–115^ Zou and colleagues found that in preterm infants exposed to antibiotics prenatally, diversity measured using the Shannon Index increased across the first 2 weeks of life but was not significantly associated with antibiotic exposure.^105^ Four studies ^106,110,113,115^ found IAP exposure significantly reduced measures of microbiome alpha diversity in infants under 1 month of age using the Shannon Index (Mazzola and colleagues ^113^ also used Simpson’s Index, Chao1, and observed species), whilst this effect was not statistically significant in two other studies.^102,114^ This trend of decreased diversity related to IAP exposure was no longer significant by 3 months of age in one study ^115^ and appeared modifiable by delivery mode in another, ^108^ where IAP with emergency cesarean delivery was associated with increased microbiota diversity at 12 months (*P* < .001) compared to vaginal delivery without IAP.

**Figure 3.**
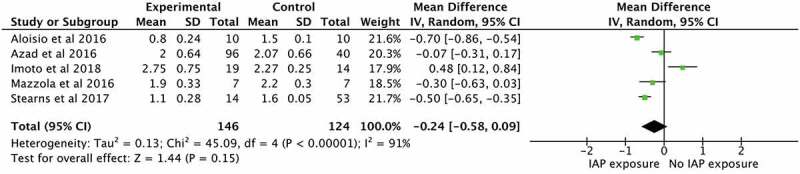
Maternal Intrapartum Antibiotic Prophylaxis (IAP) exposure in relation to infant intestinal microbiome diversity, as measured by the Shannon Index

Means and confidence intervals were pooled in a meta-analysis including five studies ^106,108,110,113,115^ which assessed the relationship between IAP exposure and alpha diversity of the infant intestinal microbiome (Figure 3). Maternal IAP exposure was associated with a nonsignificant reduction in infant α-diversity (mean difference, −0.24, 95% CI: −0.58–0.09), with high heterogeneity (I^2^ = 91%). Each study used 16S rRNA gene sequencing for microbiome analysis though the age of infants at sampling varied from 1 week to 3 months.

#### 3.4.3 Maternal pre-pregnancy BMI and gestational weight gain (GWG)

The influence of maternal pre-pregnancy BMI, ^121–126^ GWG, ^127^ or both, ^117,128–130^ on the infant intestinal microbiome was investigated in 11 observational studies. Standardised definitions of BMI were used to categorize women as having underweight (<18.5kg/m^2^), normal weight (18.5-25 kg/m^2^), overweight (25-30kg/m^2^), or obese (>30kg/m^2^).^131^ GWG was categorised as inadequate, adequate or excessive, based on the Institute of Medicine’s recommended rates of weight gain according to pre-pregnancy BMI.^132^

Associations between maternal pre-pregnancy BMI, GWG and infant microbiome composition and/or diversity were inconsistent and were modified by delivery mode in three articles.^124,126,128^ In one study including 74 neonates, maternal pre-pregnancy BMI in the overweight or obese range was associated with a significantly different gut microbial community structure (P < 0.001), enriched in the Bacteroidetes and depleted in *Enterococcus* and Proteobacteria (notably *Acinetobacter* and *Pseudomonas*), though only in infants delivered vaginally.^124^ In a small study of 30 mother-infant pairs, comprising 12 women with pre-pregnancy obesity compared to 18 women with normal weight, two-week-old infants whose mothers were overweight were associated with a significantly higher relative abundance of *Bacteroides* (P = 0.028) and lower relative abundance of Proteobacteria (P = 0.03) compared to controls.^123^ Meanwhile, in another small study (n = 42), the relative abundance of *Bacteroides* was lower in infants at one month of age whose mothers were overweight or obese prior to pregnancy (P = 0.028) or had excessive GWG (P = 0.025).^130^ Both associations were no longer significant by six months of age.^130^ Results from the New Hampshire Birth Cohort ^128^ also indicated that birth mode modified associations between pre-pregnancy BMI and the infant gut microbiome at six weeks of age; in those delivered vaginally, maternal overweight/obesity was associated with increased infant gut microbiome diversity and a higher relative abundance of 15 operational taxonomic units (OTUs), but there were no significant associations between pre-pregnancy BMI and infant gut microbiome diversity in those born via caesarean-section. Meanwhile, GWG was not associated with measures of microbial diversity or a different relative abundance of gut microbial OTUs in infants, regardless of delivery mode.^128^ When examined across the first two years of life, another study found that whilst maternal pre-pregnancy BMI and GWG affected the maternal microbiome, they were not associated with significant differences in offspring gut microbiota in composition or diversity.^129^

**Figure 4.**
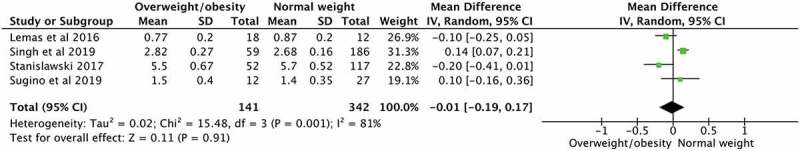
Maternal pre-pregnancy obesity in relation to infant intestinal microbiome diversity, as measured by the Shannon Index

**Figure 5.**
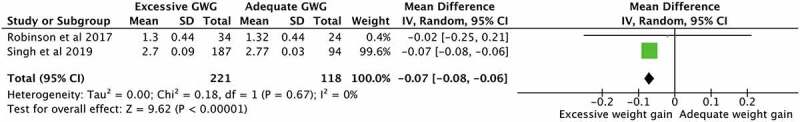
Maternal gestational weight gain (GWG) in relation to infant intestinal microbiome diversity, as measured by the Shannon Index

Associations between infant microbiome alpha diversity (Shannon Index) and maternal pre-pregnancy BMI (normal weight versus overweight or obesity) were analysed in a meta-analysis including four studies ^123,125,128,129^ (Figure 4). Maternal overweight/obesity was associated with a marginally lower Shannon Index (mean difference, −0.01, 95% CI: −0.19-0.17), with considerable heterogeneity (I^2^ = 81%). Maternal GWG and alpha diversity of the infant intestinal microbiome was also meta-analysed across two studies ^127,128^ (Figure 5). Excessive GWG was associated with a slightly lower diversity in infants compared to those whose mothers had adequate GWG (mean difference, −0.07, 95% CI: −0.08 – −0.06, I^2^ = 0%).

#### 3.4.4 Maternal diet and nutrient supplementation during pregnancy

Data exploring associations between maternal diet (using dietary questionnaires) and the infant intestinal microbiome were mixed across six included studies. Two observational studies investigated broad dietary patterns ^133,134^ and one assessed maternal fat intake.^135^ One trial explored the effect of salmon intake during pregnancy, ^136^ and two intervention studies examined maternal vitamin D supplementation.^137,138^

Maternal dietary food (salmon) or food group intake and vitamin D supplementation were not found to have significant independent impact on the infant microbiome, after adjusting for other factors including demographic characteristics (e.g. race/ethnicity, maternal education), delivery mode and/or breastfeeding status. Urwin and collegues ^136^ found that maternal consumption of salmon twice weekly was not associated with a significant effect on maternal or infant gut microbiome composition; infants in the salmon group who were formula-fed were associated with a trend toward lower bacterial counts of the *Atopobium* cluster compared to controls, though this was not observed in breastfed infants. Using dietary questionnaires, Lundgren and colleagues ^133^ reported that maternal fruit consumption was significantly associated with infant stool microbiome composition at six weeks (P = 0.028) though only in vaginally delivered, exclusively breastfed infants. Similarly, the Vitamin D Antenatal Asthma Reduction Trial (VDAART) ^134^ found that whilst a “healthy” dietary pattern charactersised by a high intake of vegetables and low intake of processed meats and deep-fried foods was associated with increased diversity (P < 0.001) and richness (P < 0.001) in the infant gut, these differences failed to reach significance in models adjusted for demographics and infant feeding mode. Another publication from the VDAART ^138^ found no association between prenatal vitamin D supplementation and infant microbial diversity at 3-6 months (P = 0.61). Limited associations between maternal vitamin D supplementation, 25-hydroxyvitamin D concentration and the infant gut microbiome were likewise observed in the KOALA birth cohort study ;^137^ with the exception of an association between vitamin D supplementation and increased counts of *Bifidobacterium* (P = 0.012) in infant stool at one month of age, no other associations were significant in adjusted models.

Conversely, Chu and colleagues ^135^ found that, independent of maternal BMI, infants whose mothers consumed high-fat diets (one SD higher than the mean fat intake) were associated with an altered meconium composition (P = 0.001) and a relative depletion of *Bacteroides* (P = 0.02) at six weeks of age, compared to controls. All infants received both breastmilk and formula at six weeks of age, except for two subjects who were exclusively breastfed. Post-hoc analysis removing these subjects did not alter the significant correlation between the reduced relative abundance of *Bacteroides* and high maternal dietary fat intake during pregnancy.

#### 3.4.5 Maternal diabetes

Four studies ^72,75,139,140^ investigated the influence of maternal diabetes mellitus (DM), including GDM, on the infant intestinal microbiome. When women with pre-existing DM, GDM and normo-glycaemia (controls) were analysed separately, Hu and colleagues ^75^ found significant overall microbiome differences between DM and GDM groups, and controls (P = 0.004 and 0.022, respectively), largely driven by relative enrichment of Bacteroidetes and depletion of Proteobacteria in women with DM. This increased relative abundance of Bacteroidetes in infants whose mothers had GDM (P = 0.029) was replicated in one recent abstract (data not shown), ^139^ but other studies ^72,140^ found inverse associations between relative Bacteroidetes abundance and both DM status (P < 0.05 for both) and maternal fasting glucose levels (P = 0.018) .^140^ At the genus level, infants whose mothers had GDM were associated with a reduced relative abundance of *Prevotella* and *Lactobacillus* (both P < 0.05), compared to controls .^140^

#### 3.4.6 Maternal mood disorders and stress

The relationship between maternal depression, anxiety or stress and the infant intestinal microbiome was explored by four observational studies .^141–144^ Maternal mood disorders were associated with changes in the infant gut microbiome; however, these were often modified by time and other environmental factors. One-week-old infants whose mothers had high depressive scores during pregnancy were associated with a lower abundance of Actinobacteria and a higher abundance of Proteobacteria compared to controls, although this difference did not persist over time .^141^ In a very large sample (n = 1,681) of four-month-old infants from the CHILD birth cohort, ^142^ infants of mothers with prenatal depressive symptoms, with or without antidepressant treatment, had higher median abundance of *Lachnospiraceae* in their gut compared to infants of mothers with few depressive symptoms, which was modified by prenatal pet ownership (P_interaction_ = 0.02). Lee and colleagues ^144^ similarly reported that whilst infants of mothers with high anxiety scores had different *Escherichia, Bacteroides, Clostridium*, and *Terrisporobacter* profiles compared to controls (data not shown), this was modified by a healthy maternal diet pattern (defined by a high intake of vegetables and fruit).

Maternal prenatal reported stress and cortisol levels were also found to be significantly associated with total microbiota composition (P < 0.01) in Zijlmans *et al* .^143^ Infants whose mothers were categorised as having experienced high cumulative stress during pregnancy (i.e., both high reported stress and cortisol concentrations) were characterised by an increased relative abundance of Proteobacterial groups, alongside reduced relative abundances of lactic acid bacteria (i.e., *Lactobacillus, Lactoccus, Aerococcus*) and Bifidobacteria, compared to controls (P values not provided).

#### 3.4.7 Maternal asthma

Two studies ^145,146^ reported on associations between maternal asthma and development of the infant intestinal microbiome. In the CHILD birth cohort study, infants whose mothers had asthma during pregnancy harboured fewer *Lactobacilli* in their gut microbiota (P = 0.02), however this was highly modified by infant sex and mother’s ethnicity .^145^ The Danish COPSAC_2010_ cohort found maternal asthma was not associated with alpha or beta diversity in the gut microbiome of 12-month-old children and described the effect of exposures across the first year of life as either triggering or protecting infants from an inherited asthma risk .^146^

#### 3.4.8 Other maternal demographics, medical conditions, and pregnancy complications

A further fifteen studies ^50,116,118–120,147–156^ analysed a variety of maternal factors in relation to the infant intestinal microbiome, including maternal ethnicity, disease, pregnancy complications and environmental exposures during pregnancy.

Lewis and colleagues ^152^ investigated maternal country of origin and found a significant difference between the intestinal microbiome of children born to mothers in Armenia and Georgia, responsible for 8.4% of the total variation in microbiome community composition between samples. Differences secondary to race-ethnicity were also found between Caucasian and African-American, ^116^ Asian, ^154^ and Latino ^150^ women in three North American studies. Compared to Caucasian, African-American race-ethnicity was associated with a more phylogenetically diverse gut microbiota in neonates (*P* = .002) and infants (*P* < .001).^116^ Groups of phylogenetically distinct bacterial genera were also differentially abundant between women recruited through CHILD and South Asian Birth Cohort (START-Canada) studies, including when adjusted for multiple confounders, including diet (*P* < .001).^154^ Whilst all studies used 16S rRNA gene sequencing, there was substantial variation in the timing of stool sampling (from 1 week to 12 months of age).

Differences in infant stool composition and/or diversity were also highlighted in relation to maternal health status during pregnancy. Compared to controls, infants born to mothers with HIV, ^147^ GBS, ^148^ atopic eczema, ^120^ or Irritable Bowel Disease (IBD) ^151^ had significant differences in gut microbiome composition and/or diversity. Maternal pregnancy complications, including chorioamnionitis, were also associated with significant differences in the intestinal microbiome of preterm infants, though the direction and significance of these associations varied. Chorioamnionitis was associated with altered alpha diversity as measured by the Shannon Index; significantly increased (*P* < .01) in one study, ^119^ though significantly reduced (*P* = .012) in another ^156^ and non-significantly lowered (*P* = .129) in the third.^50^

The remaining studies explored lifestyle, ^149^ pollution, ^153^ pet ownership ^155^ or multiple factors ^118^ during pregnancy and the infant microbiome and are outlined in Supplementary Table 1. These exposures warrant future investigation; although some studies were large, associations were often not independently associated with the infant gut microbiome and require corroboration with further studies.

### Risk of bias in included studies

As shown in Supplementary Table 1, two intervention studies ^91,92^ and two observational studies ^109,140^ (5% total included studies) were considered to be of high methodological quality as defined by the SIGN checklists. These were characterized by clearly explained research questions, inclusion/exclusion criteria, and demographics of subjects, participant randomization, and concealment procedures. Potential confounders were also considered and accounted for in statistical analyses.

The majority of included studies (83%, n = 63) were classified as being of acceptable methodological quality using the SIGN criteria. These studies included clearly defined research questions, randomized assignment of participants (where possible), and valid measurement of clinical data and the infant intestinal microbiome. However, these studies often did not clearly define concealment methodologies, and/or provide adequate detail regarding potential baseline differences between groups or confounding variables, and/or comment on cohort dropout rates.

Three trials (4%) were considered of poor quality, providing inadequate information regarding randomization, concealment, group differences, and attrition rates to allow for a higher rating.^84,85,89^ These publications were not included in any meta-analyses. Six abstracts (8%) also did not provide sufficient detail in order to warrant quality assessment.^112,121,139,141,144,151^

## DISCUSSION

The current systematic review of 20 interventions and 56 observational studies encompasses 12,770 and 4,312 mother-infant dyads, respectively, and provides a comprehensive synthesis of available data on prenatal factors affecting the infant gut microbiome. The review synthesizes current evidence on these relationships, including whether (or not) they affect the infant microbiome and consider their potential impact on infant (and/or long term) health outcomes, aligned with the DOHaD framework. To our knowledge, this is also the first review to quantify associations between maternal factors and measures of the infant intestinal microbiome, using meta-analysis where possible.

Multiple exposures, including maternal antibiotic treatment and probiotic use, diet, anthropometry, chronic health conditions (obesity, diabetes, asthma, and mood disorders), and genetic and environmental factors have been examined for their association with the infant intestinal microbiome. However, current evidence needs to be interpreted with caution, as the methodological quality of studies in the field is variable and the reproducibility, magnitude, and longevity of the effects of these exposures – within the context of a complex network of contributing factors – remain challenging to determine.

### Maternal exposures and the infant microbiome – a complex picture

#### Augmentation of the maternal microbiome via probiotic or prebiotic supplementation

There is increasing interest in the manipulation of the intestinal microbiome through the use of probiotic or prebiotic supplements during pregnancy to optimize maternal and infant outcomes. However, despite representing the exposures for which most data were available, the current review provides little support that probiotic or prebiotic supplementation during pregnancy has enduring effects on the offspring intestinal microbiome. Apart from shortly after birth, there were no significant differences between groups in the relative abundance of supplemented species or overall microbiome diversity over time.^82,85,86,93,97,94,100^ Many studies also provided probiotics to mothers *and* infants, such that the specific effect of maternal supplementation on the infant gut microbiome and/or later-life health outcomes could not be assessed.^85,92,93^ Our findings are consistent with a recently published systematic review and meta-analysis which did not find evidence to support probiotic or prebiotic use in reducing the risk of preterm birth or other adverse maternal and infant outcomes, including GDM, PPROM, and small- and large-for-gestational age (SGA, LGA).^157^

#### Suppression of the maternal microbiome via antibiotic exposure

Antibiotics are provided to approximately 25% of pregnant women and account for nearly 80% of all medications prescribed during pregnancy.^158,159^ Evidence supporting an association between early antibiotic exposure and the risk of allergy/atopy, ^160^ obesity, ^161^ diabetes, ^162^ and celiac disease, ^163^ and concerns regarding antibiotic resistance, ^164^ highlight the need for management guidelines regarding the requirement, dose, and duration of perinatal antibiotics considering the risk of altering maternal and offspring intestinal microbiota.

The current review found that maternal antibiotic exposure during delivery was associated with a modest reduction in alpha diversity in their infants, compared to unexposed controls (Figure 3). In non-obese diabetic (NOD) mouse models, prenatal antibiotic exposure has likewise been associated with significantly lower diversity using the Shannon index (*P* = .042), ^165^ and reduced bacterial diversity has been linked with an elevated risk of poorer health indicators, including obesity and inflammation.^166,167^ However, many studies in the current review found that differences in infant gut microbiome composition and/or diversity owing to antibiotic exposure were likely to disappear after only a month.^101,109,106,109,113,109,114^ The type, timing, and duration of antibiotic exposure (if collected) also varied (Figure 2). Whilst one study ^115^ found a dose–response relationship between the duration of antibiotic treatment and *Bifidobacterium* relative abundance, further research is required to strengthen the evidence for this association and the duration of effects. Additionally, whilst some studies focused on the use of IAP for specific indications, e.g., women with GBS, ^106,111,114,115^ ongoing investigation is required to understand whether the indication for antibiotic treatment is equally, or perhaps more, important than the impact of antibiotic exposure, per se. For example, routine IAP in planned cesarean section deliveries may confer substantially different risks to infants when compared to IAP treatment for other reasons (e.g., PPROM or chorioamnionitis), which may have independent effects that are modified or compounded by antibiotic exposure. Overall, robust evidence to address the impact of maternal exposure to antibiotics on infant health is lacking. Whilst some data were available to suggest that maternal antibiotic exposure presents an elevated risk for altered community assembly, it was of small effect size and the ability to clearly separate the influence of antibiotics from other factors is limited.

#### Maternal weight status

Through two meta-analyses, the current review highlights that maternal pre-pregnancy weight and GWG are modestly associated with infant intestinal microbiome diversity, with maternal overweight/obesity and excessive GWG both associated with a slightly reduced Shannon Index (Figures 3 and 4). Whilst Collado and colleagues ^130^ found numerous compositional differences in OTUs classified to genus according to maternal pre-pregnancy weight and GWG in infants at one and 6 months of age, including higher counts of *Bifidobacteria* in women with normal pre-pregnancy BMI and GWG, others found limited compositional differences in the intestinal microbiota related to maternal weight or GWG, ^129,168^ even when there were differences in the gut bacterial composition of mothers according to their weight status.

Worldwide, an estimation of nearly forty million pregnant women have overweight or obesity, placing them at greater risk of pregnancy complications that could affect their infant in the short and long terms.^169,170^ However, as our review highlights, despite differences shown in animal models, ^171^ maternal weight status is a limited predictor of maternal and infant microbiome composition, ^172^ likely secondary to the complex interplay between weight and other environmental and genetic factors. During pregnancy, intestinal bacterial populations change dramatically in composition, richness, and diversity ^70^ even though differences have been observed between obese and lean women ^73,173^ and according to GWG strata.^174^

Further investigation is needed using higher resolution microbiome analyses over longer time-courses to assess the influence of maternal perinatal weight status and GWG on the composition and diversity of her infant’s microbiome. Maternal obesity *per se* may not reliably impact the infant gut microbiome when variations in delivery mode, breastfeeding, and the transition toward family foods are considered. Future studies should also seek to determine whether aspects of the maternal diet during pregnancy mediate the effects of maternal pre-pregnancy weight and GWG on the infant microbiome to inform both weight management and nutrition recommendations for women prior to and during pregnancy.

#### Maternal diet

Evidence that suboptimal nutrition at conception and across pregnancy influences pregnancy, birth, and infant outcomes, including the prevention of neural tube defects ^175^ and impaired embryonic and fetal growth and development have been well described.^176,177^ However, in the current review, there were few eligible studies investigating the impact of diet in pregnancy, with limited outcomes related to the infant microbiome once adjusted for covariates including demographics, delivery mode, and/or breastfeeding status.^133–138^

The relationship between dietary components or patterns and infant intestinal microbiome composition and/or diversity is not well understood.^78^ Links between dietary macronutrient composition and GDM risk ^178^ and “unhealthy” dietary patterns and a higher risk of preterm birth and low birth weight ^179^ have been described. In this review, once delivery mode, demographic and other characteristics were controlled for, there were no significant associations between maternal dietary patterns and the infant gut microbiome before 6 months.^133,134^ Insufficient data were available to elucidate longer-term effects of the interaction between maternal diet during pregnancy and the offspring microbiome.

Beyond dietary patterns, other research has focussed on the relationship between gestational macronutrient intake, specifically fat, and infant outcomes. Evidence from an animal model indicated that a maternal high-fat diet induced microbiome changes in offspring which were partially correctable by a low-fat “control” diet after weaning.^180^ When extended to humans, ^135^ the neonatal gut microbiota also differed according to maternal fat intake during pregnancy, independent of maternal BMI, mode of delivery, and breastfeeding status. The stools of infants exposed to a high-fat diet (>40% total energy) during pregnancy (without significant differences in sugar or fiber intake) were characterized by a lower relative abundance of *Bacteroides* species known to contribute to modulating host metabolism and immune system development.^135^ Depleted *Bacteroides* and higher proportions of Firmicutes species abundance are associated with obesity in children ^181^ and adults, ^172^ suggestive of a mechanistic link between diet, microbiome, and altered weight maintenance consistent with the “dysbiosis” concept. However, an altered Firmicutes to Bacteroidetes ratio as a hallmark of obesity remains debated.^182^

Another area of interest has been in exploring the links between maternal diet, the intestinal microbiome, and infant immune development, and propensity to allergic diseases, including asthma, eczema, and food allergies.^183,184^ A large systematic review and meta-analysis in 2018 including 260 original studies found that fish oil supplementation during pregnancy and lactation may reduce the risk of allergic sensitization to the egg but did not find associations between other dietary exposures and allergic or autoimmune disease risk.^183^ Data were inconclusive or inconsistent for many dietary exposures therefore definitive conclusions could not be drawn.^183^ Maternal dietary intake and *Prevotella* presence during pregnancy and the relationship with offspring food allergy risk have been described recently by authors of the Barwon Infant Study.^185^ A large, dose–response relationship was found between maternal *Prevotella* in stool and a reduced risk of infant food allergy risk at 12 months. However, associations between maternal diet during gestation (fiber intake) and maternal *Prevotella* presence or offspring allergy development were not found.^185^ While previous research has suggested the release of lipopolysaccharides (LPS) from gram-negative bacteria induces inflammation associated with the progression of disease, ^186,187^ mechanistic links between maternal dietary components, maternal gut microbiome and offspring microbiota, immune development, and susceptibility toward allergic disease remain to be elucidated.

#### Moving beyond independent exposures

The current review highlights the difficulty of analyzing the contributions and/or interactions of individual prenatal exposures and the infant microbiome. Since both mothers and infants can be exposed simultaneously to many different factors (the impacts of which may depend on timing and/or dosage, maybe highly modifiable over time, are likely to be related to and/or interacting with each other, and can influence the delivery and postpartum factors), the need to consider a comprehensive, life-course approach is clear.

### 4.2 Microbiome measurement and assessment of composition and diversity

Over the past 15 years, advances in technology, especially culture-independent high-throughput sequencing technologies, have resulted in an exponential increase in research characterizing the composition and function of the microbiome and examining their associations with health outcomes.^34,35^ Simultaneously, conventions in naming, cataloging, and analyzing microbes and their properties have evolved considerably.^34^

A key finding of the current review is that the frequency and timing of stool sample collection, level of taxonomic resolution, microbiome analysis methodologies, and indices used to measure diversity vary across studies, limiting the meaningful synthesis of results. Since the intestinal microbiome is dynamic, comparison of microbiome associations with other variables must account for temporal variation and cannot be accurately compared across developmental stages, owing to transitions in diet and immune development. Some included studies collected meconium, which is likely not a distinct biological community assembled through *in situ* growth, but rather reflective of microbes from other maternal body sites via amniotic fluid or placental transfer.^120,188,189^ More than 40% of included studies were also limited by the use of fecal sampling at a single timepoint, with an average of two samples per study, representing only a snapshot of the rapidly evolving microbiota during infancy and highlighting the need for consecutive sampling in future research (Table 1 and Supplementary Table 1).

Alpha diversity was used as the primary outcome for the three meta-analyses in this review, in an attempt for a consistent measure against which study outcomes could be compared, and because diversity is often associated with a “healthy gut”.^166,167^ Alpha diversity metrics collapse the multiple dimensions of community structure to one dimension (e.g., species richness) or integrate multiple dimensions into a single number (e.g., richness and abundance in the Shannon Index).^98^ Such measures are useful to detect the presence of change but are limited in that they do not identify the nature of that change.^98^ The Shannon Index was selected as the measure of alpha diversity, since it was the most often used index across included studies. Synthesis of all available results through the inclusion of studies using different diversity indices would require a reanalysis of raw data and was beyond the scope of the review.

Finally, while most studies included in the current review applied 16S rRNA sequencing to provide an overview of the taxonomic profiles of the intestinal microbiome, a smaller number of more recent studies incorporated metagenomic techniques in their study design.^72,91,95,117,118,121,123^ Amplicon sequencing provides a comprehensive measure of composition within the resolution limits of the marker gene, whereas metagenomic samples all genes from the microbial community in a sample to provide finer taxon resolution (at the expense of sample depth) and more direct information on the diversity and function of intestinal microbiome communities.^190^ Technical changes are swiftly advancing the field, and whilst careful attention needs to continue to be applied to the challenges of analyzing microbiome data, owing to its compositional nature ^191^ and interpretation of results, ^190,192^ exploring the early life metagenomic profile will allow for enhanced understanding of the function of intestinal microbiota (and their metabolites) in the disease process prior to clinical presentation – a crucial path forward in linking the role of the microbiota in health and disease.

### Limitations

This review was constrained by the limitations of included studies and the current body of evidence. Although our adherence to a protocol registered with PROSPERO, methodological reporting in line with the PRISMA statement, and detailed data extraction allowed for a comprehensive comparison between a large number of studies, there was substantial heterogeneity of data (Table 1). Whilst useful in highlighting the diversity of tools used and areas of opportunity for further research, this heterogeneity meant many studies could not be combined in meta-analyses. Abstract publications in the past 2 years were included to address potential publication bias, though without peer review this increases the risk of inclusion of studies of poor quality. Non-English language studies were ineligible, which limited the number of included studies. Finally, paternal factors were not explored in the current review but represent an important focus of future research.

### Implications for practice and recommendations for future research

This review highlights that the effects of prenatal and postnatal exposures on the developing gut microbiome are additive, with their relative impacts difficult to isolate, such that there is a need to move beyond simple associations toward more complex analyses and with multiple measures over time, in order to understand patterns and mechanisms without mis- or over-interpretation of data.^35^ Crucially, just as questions related to the maternal transfer of microbiota cannot be reliably answered without strain-level classification, understanding the factors that influence the infant intestinal microbiome is not possible with a single timepoint and sample. Current variability in the literature pertaining to sample size, methodologies used to characterize the microbiome, and reporting standards have also contributed to inconsistent and often non-comparable results. Best-practice guidelines for microbiome analysis and reporting provide a useful starting point from which to ground future research, ^190,192^ alongside tailoring research designs, such that they are powered to reliably answer these knowledge gaps.

Future research should also consider exposures during the preconception environment, which are arguably equally important in establishing long-term health and disease risk.^193^ Whilst elements of a mother’s health during this period, including her diet, have been previously studied, ^177^ it is unknown whether the preconception microbiome is also important, how this varies across populations, and whether preconception presents a specific window of opportunity for microbial intervention.

There is a need for large, prospective studies, including: (i) broad, ethnically diverse cohorts, especially in under-represented populations and continents (Australia, Africa, Southeast Asia, and South America), (ii) both healthy and subjects with chronic disease, (iii) a variety of biological samples (including, but not limited to, the gut microbiome) and data on dietary, environmental and social determinants, and (iv) early recruitment (from preconception) and longitudinal follow-up are required in order to identify critical periods of change across the life course and to better understand mechanistic links between exposures, alterations in microbiome composition, diversity and/or function, and subsequent disease.

A core data set, including standards for methods and outcomes used to characterize the human microbiome, alongside comprehensive characterization of exposures and environments and standardized conceptual terminology, is needed in order to standardize research in the field and build the evidence base to allow for meaningful recommendations.^194^ Table 4 provides preliminary recommendations for future studies, including considerations for mothers and infants, and study methodologies. It is acknowledged that continued advancement in the field, especially in metagenomic technologies, will inevitably redefine what is known and how current unknowns are explored.

**Table 4.** Data collection and terminology considerations for pregnancy and infant microbiome studies

MATERNAL CONSIDERATIONS		
Domain	**Exposures**	**Timing**
*Demographics*	Age at study entry; Ethnicity; Socioeconomic status; Parity; Health conditions (especially allergic or immune disease)	At recruitment
*Antibiotic use*	Indication; Type; Duration of use; Number of Courses	Within 6 months of conception, during pregnancy and/or during delivery
*Probiotic use*	Type/Strain; Frequency of use; Duration of use	Within 6 months of conception and during pregnancy
*Anthropometry*	Weight; Height; BMI; GWG. Ideal: Waist-To-Hip Ratio; Body Composition (Fat Mass; Fat-Free Mass)	At conception and across pregnancy Ideal: at least thrice in pregnancy
*Dietary intake*	Dietary patterns; Nutrient intake; Dietary Diversity; Fiber intake; Dietary Supplement Intake (type, frequency and duration of use)	Within 6 months of conception and during pregnancy Ideal: at least twice in pregnancy
*Diabetes status*	Diagnosis of type I or type II diabetes mellitus prior to conception; Diagnosis of GDM; Diagnosis of Insulin Resistance; Metformin use; Insulin use	At conception and across pregnancy
*Mental health*	Stress; Self-Efficacy; Mental Illness (Depression; Anxiety; Others)	At conception and during pregnancy Ideal: at least twice in pregnancy
*Medications (e.g. PPIs, steroids)*	Indication; Type; Duration; Number of Courses	Within 6 months of conception, during pregnancy and/or during delivery
*Others as specific/pertinent to research questions, e.g. sleep, household size, social support, health literacy, air pollution, pet ownership, etc.*		As relevant to outcomes of interest
INFANT CONSIDERATIONS		
Domain	**Exposures**	**Timing**
*Demographics*	Gender; Gestational Age; Siblings (Number; Ages)	At birth
*Birth mode*	Vaginal delivery; Surgically assisted delivery; Spontaneous or Induced; Indication for cesarean delivery, if applicable)	At birth
*Antibiotic use*	Indication; Type; Duration of use; Number of Courses	At birth and any exposure across the first 1,000 days
*Probiotic use*	Type/Strain; Frequency of use; Duration of use; Indication (if applicable)	Any exposure across the first 1,000 days
*Anthropometry*	Weight; Length; Weight-for-Length Ideal: Body Composition (Fat Mass; Fat-Free Mass)	At birth, 6 months, 12 months and 24 months Ideal: whenever dietary intake is assessed
*Breastmilk or Formula feeding*	Breastfeeding; Formula-feeding; Mixed-feeding; Duration of Exclusive Breastfeeding; Total Duration of Any Breastfeeding; Total Duration of Formula-Feeding; Type of Formula (Standard; Hydrolyzed; Prebiotic-Containing)	From birth and across the first 1,000 days, at least four times (e.g. 0–3 months, 4–6 months, 8–12 months and 12–24 months)
*Solid food intake*	Age at Introduction to Solids; Dietary patterns; Nutrient intake; Dietary Diversity; Fiber intake; Texture	From 4 to 24 months, at least three times (e.g. 4–6 months, 8–12 months and 12–24 months)
*Medications*	Indication; Type; Duration; Number of Courses	At birth and any exposure across the first 1,000 days
*Others as specific/pertinent to research questions, e.g. neurocognitive development, sleep, household size, air pollution, pet ownership, etc.*		As relevant to outcomes of interest
METHODOLOGICAL CONSIDERATIONS		
Domain	**Outcomes/considerations**	
*Overall design*	Recruitment methods and dropout rates clearly described; Comparisons made between participants and those lost to follow up, by exposure status; Main potential confounders are identified and accounted for in study design and analysis; Randomization and concealment occur where possible.	
*Terminology*	Concepts or terms used to describe the gut microbiome should be consistent across studies.	
*Microbiome analysis*	Use of culture independent methodology (e.g. 16S rRNA gene sequencing, metagenomic sequencing); Measures of composition (e.g. total bacterial counts, relative abundance); Measure of diversity (e.g. Shannon and Simpson indices) and richness (e.g. Chao1); Appropriate OTU classification (to amplicon sequence variants, or metagenomic strains); Use of compositional data analytical approaches; Clear reporting of methodologies used and limitations of same.	
*Sampling frequency*	Longitudinal measurement across the first 1,000 days, with most infant samples after 2 weeks, e.g., Mothers in early pregnancy (0–12 weeks); Mothers in late pregnancy (32–40 weeks); Infants at 1–3 months (prior to introduction of solids); Infants at 4–6 months (when solids have been introduced); Infants at 9–12 months (increasing dietary diversity and complexity, possible weaning formula and/or breastfeeding, introduction of other milks).	

BMI = Body Mass Index; GWG = Gestational Weight Gain; GDM = Gestational Diabetes Mellitus; PPI = Proton Pump Inhibitor; OTU = Operational Taxonomic Unit.

## CONCLUSIONS

The current systematic review with meta-analysis provides a comprehensive synthesis of available data on prenatal factors affecting the infant gut microbiome, gaps in existing knowledge, and opportunities for future research. Whilst there is strong interest in how microbial communities are established throughout gestation, infancy, and childhood, and how they alter in response to innate and environmental exposures, there is a lack of consistency in methodologies concerning recruitment, follow-up, clinical data collection, stool sampling, and microbiome analysis, necessary for robust comparison and synthesis of results. Standardization in research investigating associations between modifiable maternal exposures and the offspring intestinal microbiome will allow for the enhanced synthesis of research findings, understanding of the mechanisms underpinning them, and the development of practical solutions to complex health challenges related to the microbiome, likely beginning well before birth.

## Supplementary Material

Supplemental Material
